# Analysis of Potent Odour-Active Volatile Thiols in Foods and Beverages with a Focus on Wine

**DOI:** 10.3390/molecules24132472

**Published:** 2019-07-05

**Authors:** Liang Chen, Dimitra L. Capone, David W. Jeffery

**Affiliations:** 1 Department of Wine and Food Science, The University of Adelaide (UA), PMB 1, Glen Osmond, SA 5064, Australia; 2 Australian Research Council Training Centre for Innovative Wine Production, UA, PMB 1, Glen Osmond, SA 5064, Australia

**Keywords:** derivatisation, sample preparation, gas chromatography, high performance liquid chromatography, mass spectrometry, untargeted identification, targeted quantitation, matrix effect, stable isotope dilution assay

## Abstract

Certain volatile thiols are some of the most potent odour-active molecules that are found in nature. Thiols play significant roles in the aroma qualities of a range of foods and beverages, including wine, with extremely low odour detection thresholds (nanogram per litre range). A fundamental understanding of their formation, fate, and impact essentially depends on the development of suitable analytical methods. The analysis of volatile thiols in foods and beverages is a challenging task when considering (1) the complexity of food and beverage matrices and (2) that thiols are highly reactive, low molecular-weight volatiles that are generally present at trace to ultra-trace concentrations. For the past three decades, the analytical evaluation of volatile thiols has been intensively performed in various foods and beverages, and many novel techniques related to derivatisation, isolation, separation, and detection have been developed, particularly by wine researchers. This review aims to provide an up-to-date overview of the major analytical methodologies that are proposed for potent volatile thiol analysis in wine, foods, and other beverages. The analytical challenges for thiol analysis in foods and beverages are outlined, and the main analytical methods and recent advances in methodology are summarised and evaluated for their strengths and limitations. The key analytical aspects reviewed include derivatisation and sample preparation techniques, chromatographic separation, mass spectrometric detection, matrix effects, and quantitative analysis. In addition, future perspectives on volatile thiol research are also suggested.

## 1. Introduction—Importance of Thiols to the Aroma of Foods and Beverages

**Figure 1:**
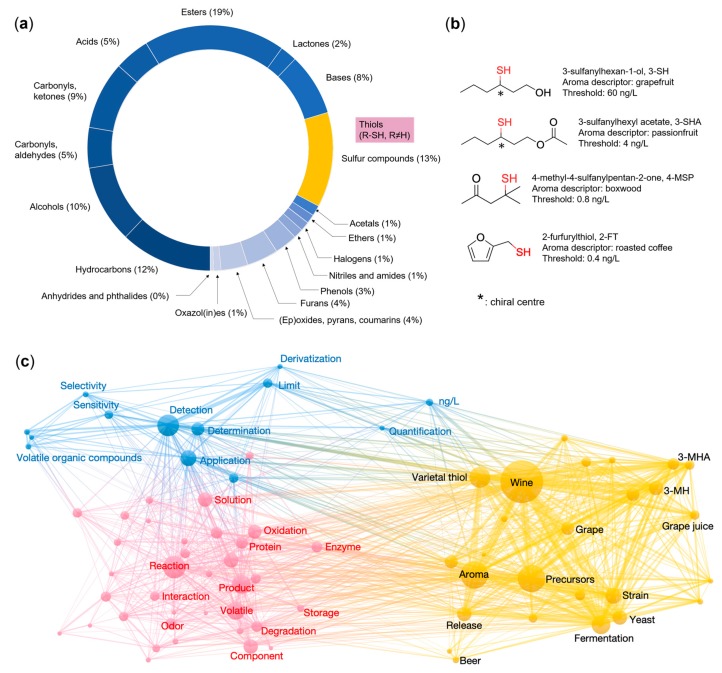
(**a**) Doughnut chart showing the relative percentages of volatile sulfur compounds identified in foods according to Volatile Compounds in Foods database [1], where each segment represents one chemical category of volatiles; (**b**) Examples of chemical structure, aroma descriptor, and ODT of some of the most studied volatile thiols in wine [4]; and, (**c**) Bibliometric map of volatile thiol research visualised from a total of 395 publications (from 1990–2019) retrieved from Web of Science Core Collection using “Volatile Thiols” as keyword. Literature analysis and graph construction by VOSviewer [5]. Note the abbreviations 3-MH and 3-MHA used in panel (**c**) are in keeping with much of the earlier literature; however, the IUPAC names (i.e., sulfanyl prefix instead of mercapto) are used in this review and abbreviated as 3-SH and 3-SHA.

Aroma is inarguably one of the most important quality aspects for any food or beverage product, with unique and characteristic aromas being attributed to a large range of volatile compounds with various physico-chemical properties. At the time of writing this review, a commercial database, Volatile Compounds in Food, had compiled a total of 9514 volatile components that were identified in natural and processed food products from published literature data [1], and the list continues to grow. Amongst the vast numbers of volatiles in the database, volatile sulfur compounds (VSCs, sulfur-containing volatiles) are the second largest category just after volatile esters, and they represent around 13% of total volatiles (Figure 1a) [1]. VSCs play an important role in the aromas of foods and beverages, not only because of their broad presence, but also for their significant sensory contributions due to concentrations that are well above their low odour detection thresholds (ODT) [2,3].

Volatile thiols, historically known as mercaptans and consisting of the structure R–SH, are a sub-category of VSCs that are of particular interest, because they have some of the lowest ODTs (ng/L and lower) of any volatile compound identified in nature [6]. Such potent volatile thiols have been in the spotlight of aroma research and they are frequently regarded as “potent” [7], “key aroma” [8], “aroma-active” [9,10], or “aroma-impacting” [11] odorants. Some of the most famous examples in foods and beverages include 3-sulfanylhexan-1-ol (aroma descriptor: grapefruit, ODT: 60 ng/L) and 3-sufanylhexyl acetate (aroma descriptor: passionfruit, ODT: 4 ng/L) in wine [6], 4-methyl-4-sulfanylpentan-2-one (aroma descriptor: boxwood, ODT: 0.8 ng/L) in beer [12] and wine [6], and 2-furfurylthiol (aroma descriptor: roasted coffee aroma, ODT: 0.4 ng/L) in coffee [13] (Figure 1b).

From a total of 395 publications (1990 to 2019, using “Volatile Thiol” as the search keyword) retrieved from Web of Science Core Collection and visualised by a network approach, it was obvious that research on volatile thiols had been focusing on “detection” and “reaction”, and the majority of publications had emerged from the field of “wine” research (Figure 1c). Indeed, much progress on the development of analytical methods for untargeted identification and targeted quantitation for volatile thiols has been achieved in wine [4]. As such, this review emphasises the field of wine research and particularly covers literature that is dedicated to developing analytical methods. The analytical challenges and requirement for volatile thiol analysis in the context of wine, foods, and other beverages are first presented, followed by strategies that were developed to address those analytical challenges. Sample preparation techniques (selective extraction with metal ions, derivatisation), chromatographic separation, mass spectrometric detection, quantitative analysis with stable isotope dilution assay (SIDA), and matrix effects have been reviewed. Finally, future trends and directions for volatile thiol research have been proposed. However, exhaustive occurrences, sensory interactions, chemical synthesis and biogenesis, and other unmentioned aspects of thiols in foods and beverages are considered as beyond the scope of this review.

## 2. Analytical Challenges and Requirements

The analysis (either untargeted identification or targeted quantitation) of volatile thiols in foods and beverages has always been a challenging task due to two reasons:

- matrix complexity;
- properties of thiols.

Volatile thiols can be found in many foods and beverages, including wine, beer, cheese, olive oil, coffee, fruit, meat, and vegetable [14]. On one hand, these foods and beverages possess drastically different matrices (animal or vegetal, fermented or unfermented, liquid or solid, aqueous or lipid, etc.) and, on the other, the matrices are compositionally complex (containing many other volatile and non-volatile metabolites). Such complexity and diversity in matrices pose analytical challenges for developing suitable and efficient analytical methods [15]. Potent volatile thiols are highly unstable small molecules that are present at extremely low abundances with diverse chemical structures. The sulfhydryl (–SH) group in thiols is one of the most reactive functional groups found in natural organic matter [16]. As such, thiols are prone to oxidation, isomerisation, and rearrangement [17]. The highly active –SH group can cause chromatographic separation difficulties even when thiols are well preserved throughout extraction, such as peak tailing during analysis by gas chromatography (GC) [18]. Apart from the instability, volatile thiols differ in chemical structure, with the majority containing either acid (–COOH), alcohol (–OH), aldehyde (–CHO), ester (–OC(O)–), ether (–O–), and/or aromatic ring functional group(s), with only a small portion belonging to aliphatic thiols [14]. Those additional functional groups should be taken into consideration at an early stage of method development in order to minimise their modification. For instance, the analysis of thiol acetates should avoid the occurrence of acetate hydrolysis during sample preparation and analysis [19]. Besides structural diversity, some thiols are also characterised with chirality, owing to the carbon bearing the sulfur atom (Figure 1b), which gives a pair of thiol enantiomers. As with any enantiomer pairs, enantiomeric thiols are of almost identical physical and chemical properties, but they often differ in aroma quality and ODT [4]. The separation of enantiomers has always been a complex task as there is no golden rule to predict chiral separation. Lastly, thiols are generally found at trace to ultra-trace concentrations and, in many cases, at part per trillion levels. Such extremely low abundances require the careful consideration of effective sample isolation and enrichment steps, and sensitive detection techniques.

An ideal analytical method for thiol analysis should be fast, simple, reliable, robust, green, sensitive, and cost-effective. For analytical methods that are dedicated to screening/discovering new volatile thiols, analytical information that is provided by the methods should be sufficient for identification. As for quantitative methods, limit of detection (LOD, ideally below ODT), matrix effects, repeatability, precision, and accuracy are among the important factors.

## 3. Thiol Isolation—Extraction and Derivatisation

Due to the analytical challenges previously mentioned, particularly the instability of volatile thiols, but also their low abundance, efficient isolation using the routine extraction techniques employed for aroma analysis is hard to achieve. This reflects on the fact that many of the established thiol isolation methods have combined rather sophisticated forms of sample pre-treatment, specific extraction, derivatisation, clean-up, and enrichment (concentration). From the methods that are summarised in Table 1, it is evident that the isolation of volatile thiols from wine (and similar applications in foods and other beverages) is an evolving process that has advanced from traditional, time-consuming methods to better-sequenced and much simplified procedures. Although being proposed in various formats, the currently available thiol preparation methods can generally be categorised into three main groups:

- non-specific extraction, e.g., straightforward application of headspace solid-phase microextraction (HS–SPME), solid phase extraction (SPE), liquid-liquid extraction (LLE), purge and trap (P+T), and vacuum distillation;
- selective extraction with metal ions (e.g., Hg^+^ or Ag^+^);
- derivatisation (coupled with LLE, HS–SPME, SPE, or gas purge microsyringe extraction (GP–MSE)).

As the non-specific extraction techniques for thiol isolation are principally similar to those that are commonly applied for other volatile compounds, selective extraction and derivatisation-based methods have been selected as the focus in this review.

### 3.1. Selective Extraction with Metal Ions

Thiol-specific extraction methods are based on the strong affinity between thiols and metal ions, such as mercury (Hg^+^) and silver (Ag^+^). In fact, the word mercaptan, which is the historical synonym for thiol, arose from the Latin term *cercurium captans*, which means mercury-seizing [20]. Organomercurial compounds have long been applied for thiol-specific extraction (Table 1, Entries 1–4) [21,22] by reversibly binding with thiols and forming stable Hg-thiol complexes (mercaptides). The thiol moiety can then be replaced from Hg-thiol complexes by an excess of other thiol (e.g., glutathione [21], cysteine [22], or d,l-dithiothreitol [23]) during an elution step, which releases the thiols of interest. Specific organomercurial compounds that have been used in this manner include 4-hydroxymercuribenzoate (usually referred to as *p*-hydroxymercuribenzoate and abbreviated *p*-HMB) [21,22,24], phenylmercuric chloride [25], and 4-aminophenylmercuric acetate [23]. These agents efficiently and selectively bind thiols; for instance, the reaction between *p*-HMB, the most popular organomercurial reagent, and thiols in wine requires less than 90 s, and, more importantly, *p*-HMB does not react with thioesters, sulfides, or disulfides [21].

Pioneered for wine with the identification of varietal aroma compounds that are associated with box tree odour, the early developed *p*-HMB extraction method (Table 1, Entry 1) requires pH adjustment on a large volume of sample (1000 mL), followed by LLE with organic solvent prior to thiol extraction with aqueous *p*-HMB solution [21]. While using this extraction method, 4-MSP was identified for the first time in the plant kingdom (a Sauvignon wine). For quantitation purposes that require cleaner thiol extracts, a strong anion exchange column was introduced as a clean-up step after the extraction of thiols from the organic phase with *p*-HMB solution, prior to eluting with cysteine solution to release the thiols (Table 1, Entry 2) [22]. This approach has been modified and used for thiol extractions in beer [12] and cheese [26]. One limitation of applying this extraction method is that it requires pH adjustment at several points (in the raw sample, during extraction, and in pooled extracts [22]), and the high pH conditions could induce thiol oxidation or unwanted changes in the sample matrix, such as the formation of quinones that can react with thiols. Therefore, the *p*-HMB extraction method has been modified to avoid tedious pH adjustment of wine by using a Tris buffer solution (pH > 7) [24].

*p*-HMB extraction procedures are quite time consuming due to the need for pH adjustment and clean-up, and, more importantly, thiols are prone to oxidation during the laborious sample preparation steps, which could influence their quantification. Affinity chromatography with Affi-Gel 501 (formed from Affi-Gel 10 by treatment with 4-aminophenylmercuric acetate) was developed for thiol extraction from extracts prepared by LLE to simplify the selective extraction step (Table 1, Entry 3) [25]. Affinity chromatography with Affi-Gel appears to be less time-consuming when compared to the previously developed *p*-HMB protocol, but it demonstrates lower recovery rates (e.g., for 4-MSP, 38% vs. 75%–80%). This approach is possibly more suited to thiol discovery, with a similar affinity chromatography approach having been used to screen for volatile thiols in various fruits and wines [23]. Another slight limitation is that Affi-Gel 501 needs to be prepared in-house, and these approaches still involve intense extraction and concentration steps (more problematic for routine quantitation than for thiol screening).

Another selective extraction method is based on the high affinity between thiols and Ag^+^ (Table 1, Entry 5) [28]. Using commercially available Ag^+^ based SPE cartridges (Meta-Sep IC-Ag), volatile thiols in the organic extracts of beer and hops were retained and then eluted with thioglycerol in CH_2_Cl_2_ [28]. Ag^+^ extraction is advantageous when compared to Hg^+^, because it avoids the use of toxic mercury and the SPE cartridges can be commercially obtained. However, the Ag^+^ extraction procedure is still somewhat complicated, with the LLE extraction of volatiles, and multiple clean-up and concentrating steps after SPE. However, from purely an extraction viewpoint, selective extraction methods have high reaction efficiency, selectivity, and permit the recovery of thiols for analysis in their unmodified form. This is particularly useful for GC–Olfactometry (GC–O) screening for new thiol odorants, and it has enabled the discovery of many important volatile thiols in wine [21,22,24], tea [46], hop extracts [12], and beer [12,47]. On the other hand, the drawbacks of these extraction approaches are obvious: large amounts of sample and solvent are required for the preparation of volatile extracts; procedures are lengthy and time-consuming; final concentrated thiol distillate/extracts are in their original sulfhydryl form, which can cause reaction, separation, and detection issues; in the case of *p*-HMB, handling highly toxic organomercurial compounds poses significant health and environmental risks.

### 3.2. Derivatisation Approaches

**Figure 2:**
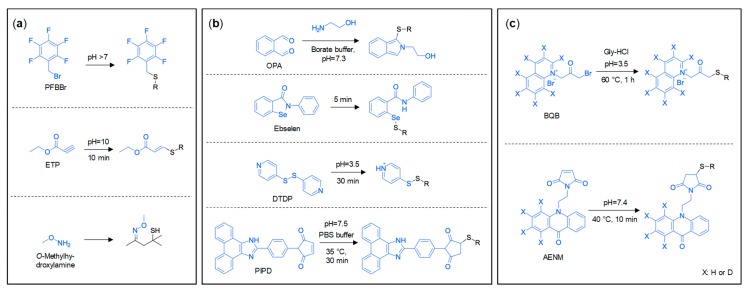
Derivatisation reagents and reactions of volatile thiols in wine, foods, and other beverages for (**a**) gas chromatography (GC) analysis, (**b**) liquid chromatography (LC) analysis, and (**c**) LC with stable isotope labelled derivatisation reagents. PFBBr: 2,3,4,5,6-pentafluorobenzyl bromide; ETP: ethyl propiolate; OPA: *o*-phthaldialdehyde; ebselen: 2-phenyl-1,2-benzisoselenazol-3(2H)-one; DTDP: 4,4′-dithiodipyridine; PIPD: 1-(4-(1*H*-phenanthro[9,10-d]imidazol-2-yl)phenyl)-1*H*-pyrrole-5-dione; BQB: ω-bromoacetonylquinolinium bromide; AENM: acridone-10-ethyl-*N*-maleimide.

The adaptation of derivatisation for more selective, efficient, and simplified isolation procedures and/or stabilisation of thiols has been the major development in thiol isolation. These approaches are designed to improve the sensitivity of instrumental analyses, because, after derivatisation, volatile thiols are easier to be extracted, chromatographed, and detected. On one hand, derivatisation intends to block the sulfhydryl group (or mask a carbonyl group in the case of 4-MSP, for example) and the formed thiol derivatives are chemically stable for isolation, as well as thermally stable for GC analysis. On the other hand, introducing a substituent means that thiol derivatives exhibit greater hydrophobicity, less polarity, and/or stronger proton affinity, which leads to better liquid chromatography (LC) separation and signal enhancement for mass spectrometry (MS)-based detection [48]. When selecting suitable derivatisation reagents, the factors to consider include reaction specificity and efficiency, matrix compatibility, required sample manipulation, introduction of interferences, and whether it occurs before or after the extraction of analytes. Figure 2 shows common derivatisation reagents and related reaction conditions that are proposed for volatile thiols analysis in wine, foods, and other beverages, and categorised into those for GC analysis (Figure 2a), or LC analysis with conventional (Figure 2b) or stable isotope labelled (Figure 2c) derivatisation reagents.

#### 3.2.1. Derivatisations for GC Analysis of Thiols

As seen in Table 1 (Entries 6–14), 2,3,4,5,6-pentafluorobenzyl bromide (PFBBr), ethyl propiolate (ETP), and *o*-methylhydroxylamine have been used as derivatisation reagents for GC-based thiol analysis. PFBBr and ETP both react with the sulfhydryl group, whereas *o*-methylhydroxylamine derivatises the carbonyl group in 4-MSP (forming a methoxime). After derivatisation(s), extractions can be conducted in combination with modern extraction techniques such as HS–SPME, SPE, or stir bar sorptive extraction (SBSE), in contrast to the traditional LLE or affinity chromatography normally practised in selective extractions with Hg^+^ or Ag^+^ (although LLE, in particular, may still feature along with derivatisation).

PFBBr (Table 1, Entries 6–11) is frequently used as a derivatisation reagent for thiols, due to the bromide atom being particularly susceptible to nucleophilic substitution by thiols in the presence of base, and the obtained PFBBr thiol derivatives offer desired properties, not only by stabilising the thiol, but also with regard to electron-capturing abilities and MS detection [18]. The derivatisation of volatile thiols in wine with PFBBr has been evaluated in various formats: automated headspace on-fibre derivatisation [18], derivatisation in organic solvent system [29] or aqueous phases [32] followed by HS–SPME, in-cartridge SPE derivatisation [30], and HS–SPME coupled with SPE [31]. The SPME on-fibre derivatisation (Table 1, Entry 6) is fast, automated, and solventless. A polydimethylsiloxane/divinylbenzene (PDMS/DVB) SPME fibre is exposed in sequence to the vapours of tributylamine (5 min), PFBBr solution (5 min), and then pre-incubated wine sample (containing ethylenediaminetetraacetic acid, salt, and internal standard (IS)) for extraction for 10 min at 55 °C [18]. This approach provided convenience and less potential interferences by using an autosampler and HS-SPME [18], but the linear ranges for the studied thiols were not very wide (and extremely narrow for 2-methyl-3-furanthiol, 2-MFT) and only two thiols (2-FT and 3-SHA) out of five were able to be analysed with this method. To improve the procedure, three conditions (two-phase liquid-liquid system, two-phase liquid-liquid system with a phase transfer catalyst, and two-phase liquid-solid system) were evaluated for wine and PFBBr derivatisation was finally conducted in a homogeneous organic solvent (benzene) system, based on relatively higher derivatisation yields and lower extraction of polar compounds from wine (Table 1, Entry 7) [29]. Apart from switching from on-fibre derivatisation to a homogeneous organic solvent system, 1,8-diazabicyclo[5.4.0]undec-7-ene (DBU) was used as a non-nucleophilic base to better deprotonate thiols (enhancing reactivity) and enable a decrease in the amount of PFBBr, which lessened the chance of PFBBr carryover. After comprehensive optimisation, the method provided larger linear ranges than SPME with on-fibre derivatisation [18], but it was still unable to achieve consistent results for 2-MFT and required the use of the carcinogenic solvent benzene [29]. Subsequently, PFBBr derivatisation (after carbonyl derivatisation) in an SPE cartridge was suggested for volatile thiols in wine (Table 1, Entry 8) [30]. This protocol involved multi-step washings, derivatisation reaction, and the elution of PFBBr derivatives, but it still suffered from interferences regardless of the improved clean-up with SPE. This was ultimately compensated for by adapting to a method involving SIDA [30]. A method with methoximation and PFBBr derivatisation in combination with both SPE and HS–SPME has been developed, also using SIDA, to further eliminate matrix effects (Table 1, Entry 9) [31]. After methoximation (for 4-MSP) [30], the wine sample was adjusted to pH 7 and derivatised in-cartridge with PFBBr and DBU, and then eluates from SPE were evaporated and sampled by HS-SPME [31]. The change of pH was necessary to eliminate interferences that were not retained by SPE at higher pH [31].

The above-mentioned PFBBr derivatisation approaches were followed by GC with chemical ionisation (CI) and MS detection in negative ion mode, instead of the more routinely available electron ionisation (EI) with MS detection in positive ion mode. As such, the PFBBr derivatisation methods were investigated for thiol analysis in wine while using GC with EI–MS, again in combination with HS–SPME of the derivatives [32,33]. Focusing on 3-SH, an extraction was proposed that involved LLE with pentane and back-extraction into cold aqueous NaOH, followed by direct PFBBr derivatisation (Table 1, Entry 11) [32]. This method showed that the analysis of a PFBBr thiol derivative could be achieved by EI–MS, although the sample volume (100 mL) was much larger than that required for NCI-MS detection, and only 3-SH was assayed (albeit at levels below its ODT) [32]. The method was improved by applying extractive arylation with PFBBr but still using GC–EI–MS (Table 1, Entry 12) [33]. The approach, which included 3-SHA and 4-MSP, along with 3-SH, employed PFBBr derivatisation of thiols in 40 mL of wine (pH adjusted to 12) with the simultaneous extraction of derivatives into pentane-diethyl ether. The extracts were dried, reconstituted, and subjected to HS-SPME [33]. The improved analytical performance, when compared to the previous method for 3-SH alone [32], was proposed to result from the removal of interferences, more optimal conditions for derivatisation or HS-SPME sampling, or having fewer steps that contribute to analyte losses during extraction [33]. In comparison to selective *p*-HMB extraction and the analysis of free thiols, the suggested PFBBr derivatisation-based methods for thiol extraction and analysis (either for CI–MS or EI–MS) have significantly lower sample volume and solvent consumption (especially with HS–SPME), less sample preparation steps, and no requirement for organomercurial compounds. However, the overall extraction processes are still lengthy and complicated (e.g., pH adjustment and multiple steps), and PFBBr is not entirely without safety concerns.

ETP is another reagent that has been investigated for the derivatisation of thiols (Table 1, Entries 12 and 13) in wine [19], beer [34], hops [34], and wort samples [34]. In the case of wine analysis, ETP rapidly reacts with thiols at basic pH (10 min), and, following an optimised SPE step, the ETP derivatives can be analysed by GC–MS (Table 1, Entry 12) [19]. The ETP-based method has further simplified the approach to thiol derivatisation when compared to *p*-HMB and PFBBr methods, but still requires pH adjustment of wine (not a trivial undertaking). The main shortcoming was the lack of sensitivity for 4-MSP in real wine samples as a result of poor derivatisation [19]. Nonetheless, ETP has also been evaluated in combination with SBSE for the analysis of 3-SH, 3-SHA, and 4-MSP in beer, hops, and wort samples (Table 1, Entry 13) [34]. The SBSE procedure is relatively simple to conduct and it requires less solvent, but the disadvantages included the need for pH adjustment of samples, that a single SBSE of derivatives required more than 3 h, that stir bars needed to be conditioned before use and reconditioned after each use, and the need for a thermal desorption unit (TDU) [34].

Finally, there is *o*-methylhydroxylamine for the derivatisation of a carbonyl group as a methoxime. Aside from its use for masking the keto functionality of 4-MSP to facilitate thiol derivatisation with PFBBr (Table 1, Entries 8 and 9), *o*-methylhydroxylamine derivatisation has been employed in an automated SIDA HS–SPME procedure for the analysis of 4-MSP at sub-ODT concentrations (Table 1, Entry 14) [35]. The procedure creates specific higher mass fragments that facilitate MS detection when compared to the natural analyte (but using positive CI rather than the more routine EI), and it requires a small volume of sample (3 mL) and less sample preparation as compared to ETP [35]. However, despite being easier to undertake and very sensitive, the procedure is only applicable to 4-MSP, and it is complicated by the fact that two derivative isomers are formed from a single analyte, with the favoured (*E*)-isomer being selected for quantitation [35].

#### 3.2.2. Derivatisations for LC Analysis of Thiols

The abovementioned specific extraction and derivatisation approaches that involve Hg^+^, Ag^+^, PFBBr, and ETP are for GC based systems. Emerging some time after many of the GC methods were developed, derivatisation-based procedures that were designed for LC analysis are perhaps one of the most important developments in thiol isolation. In comparison to their GC counterparts, the suggested derivatisation protocols for LC analysis of volatile thiols have tended to simplify the overall extraction protocol and offer excellent sensitivity in a variety of matrices. Derivatisation reagents applied for LC-based analysis of thiols (Table 1, Entries 15–19) include conventional reagents, like *o*-phthaldialdehyde (OPA) [36], 2-phenyl-1,2-benzisoselenazol-3(2H)-one (ebselen) [37,38,39,40], 4,4′-dithiodipyridine (DTDP) [7,41,42], and 1-(4-(1*H*-phenanthro[9,10-d]imidazol-2-yl)phenyl)-1*H*-pyrrole-5-dione (PIPD) [45], as well as stable isotope labelled reagent pairs d_0_/d_7_-ω-bromoacetonylquinolinium bromide (d_0_/d_7_-BQB) [43,49] and d_0_/d_4_-acridone-10-ethyl-*N*-maleimide (d_0_/d_4_-AENM) [44]. The reagents readily react with the sulfhydryl group, and derivatisation reactions are often performed in various formats, such as in conjunction with LLE, SPE, or GP–MSE prior to LC analysis, to assist with sample clean-up and enrichment.

OPA was considered for thiol derivatisation in white wine [36] due to the reactivity of the dialdehyde functionality with amino acids and other nucleophiles, including thiols [50], thus beginning the exploration of LC-based approaches for the analysis of volatile thiols in wine (Table 1, Entry 15). Derivatisation with OPA in the presence of ethanolamine under basic conditions is rapid (5 min at room temperature in borate buffer), but 4-MSP was unable to be derivatised and the pre-derivatisation sample preparation steps are rather complicated. Briefly, wine has to be treated with potassium metabisulfite and polyvinylpolypyrrolidone, followed by pH adjustment and reaction with borohydride, and then LLE with CH_2_Cl_2_ (possibility of forming an emulsion) and sample concentration steps prior to derivatisation [36]. Moreover, the OPA-thiol derivatives were unstable, even when stored at −80 °C, and their rapid and significant degradation would ultimately lead to a loss of sensitivity and inaccurate quantitation [36].

The Se-N-containing reagent 2-phenyl-1,2-benzisoselenazol-3(2H)-one (ebselen) selectively and efficiently reacts with thiols by the cleavage of Se-N bond and formation of an Se-S bond with the –SH group [51]. Ebselen has been used to derivatise a range of thiols (such as those in Figure 1b) in various matrices (lipid: olive oil [37]; hydroalcoholic: wine [38], beer [38]; aqueous: brewed coffee [39]; organic extract: roasted coffee [40]) (Table 1, Entry 16). The proposed ebselen derivatisation approaches are fast, single-step derivatisation/extraction (~ 1 min), with some slight variations in initial sample preparation, solvent choice, solvent volumes, and workup steps, depending on sample matrices. In general, a suitable solvent containing ebselen (or with ebselen introduced separately) is added to the solid or liquid sample. After vortexing for a short period of time, the organic phase is collected, concentrated, and the residue is re-dissolved for analysis (or analysed directly without concentration) [37,38,39,40]. The major advantages of these approaches when compared to aforementioned derivatisation procedures is they are much less complicated and they employ mild conditions, although they still require the handling of samples under N_2_, and the instruments are all high resolution mass spectrometers as opposed to the more common triple quadrupole. As a slight aside, these derivatives are claimed to enhance ionisation and improve the signal response due to the ease of ionisation and the positive charge gained by the nitrogen. However, the nitrogen in ebselen derivatives is an amide (weak acid) and not an amino nitrogen, so positive ionisation mode would rely on the protonation of the carboxamide oxygen [52].

Thiol derivatisation with DTDP has also been developed for wine analysis (Table 1, Entry 17) [7], due to its high derivatisation ability of sulfhydryl groups at acidic pH [53], whereby DTDP specifically and rapidly directly reacts with thiols in the natural wine pH range [7]. Avoiding pH adjustment throughout derivatisation and extraction is deemed to be an important point, and, when coupled with conventional SPE for clean-up and enrichment of derivatives, this procedure provides a relatively simple approach that affords the desired sensitivity for analysis of 3-SH, 3-SHA, 4-MSP, BT, and 2-FT [7]. The flexibility of the DTDP derivatisation and extraction method has been demonstrated in the chiral analysis of enantiomers of 3-SH and 3-SHA in wine [41], and in a refined form (no concentration after SPE) while using convergence chromatography for wine analysis [42]. The additional advantages of DTDP derivatisation include the formation of stable and easily ionisable derivatives (due to the pyridine moiety) that are ideal for electrospray ionisation (ESI), and, to a lesser extent, the inclusion of a chromophore, which may be useful for samples with high levels of thiols (3-SH in particular), although this was not tested. As a bonus, DTDP is a non-hazardous chemical and is safer to deal with than some other reagents, especially *p*-HMB or PFBBr.

More recently, 1-(4-(1*H*-phenanthro[9,10-d]imidazol-2-yl)phenyl)-1*H*-pyrrole-5-dione (PIPD) has been demonstrated as a new derivatisation reagent for volatile thiols (Table 1, Entry 20). The maleimide moiety in PIPD can rapidly react with thiols to form derivatives that are stable (4 °C for at least three days) and detectable by HPLC–fluorescence (with atmospheric pressure chemical ionisation (APCI)-MS used for identification) [45]. Thiol extraction was performed with GP–MSE (N_2_ at 2.5 mL/min for 30 min at 190 °C) in a customised apparatus prior to derivatisation (10 min at 35 °C in phosphate buffered saline, pH 7.5) [54]. After extraction and derivatisation, the mixtures were diluted with methanol (MeOH), filtered, and directly injected [45]. This proposed extraction and derivatisation methodology is simple and fast, and the analytical method is precise and sensitive, but the approach requires a customised gas purge chamber and the sample is kept at a high temperature for 30 min, which seems unlikely to be applicable to liquid samples, such as wine or beer. In addition, PIPD has to be synthesised, as opposed to other commercially available reagents.

Whether for LC or GC, the derivatisation examples that have been mentioned so far require the use of reference standards and internal standards to establish the calibration curves for quantitative analysis. In many cases, the reference standards or internal standards (particularly deuterated internal standards) are not commercially available, or they are expensive to acquire or non-trivial to synthesise. These concerns can be somewhat simplified by the use of stable isotope labelled derivatisation-based methods [43,44,49], and, when considering that the derivatisation of volatile thiols appears to be essential for food and beverage analysis by LC, introducing stable isotope labelled derivatisation does not add any extra sample processing steps. Reagents for stable isotope labelled derivatisation can not only enhance the stability and detectability of thiols, just like the conventional reagents, but also provide advantages in facilitating untargeted identification and targeted quantitation based on the characteristic mass differences between the unlabelled/labelled derivative pair that are easily distinguishable by MS [55]. Stable isotope labelled derivatisation reagent pairs that have been used for thiol analysis in beverages include d_0_/d_7_-ω-bromoacetonylquinolinium bromide (d_0_/d_7_-BQB) for beer (Table 1, Entry 18) [43,49] and d_0_/d_4_-acridone-10-ethyl-*N*-maleimide (d_0_/d_4_-AENM) for wine (Table 1, Entry 19) [44]. Both of the reagents utilise a reactive group (bromide for BQB, maleimide in AENM), an ionisable group, and an isotopically labelled group in one of the pairs. BQB derivatisation consists of rather simple sample preparation steps, which only involve drying and derivatisation (60 °C, 60 min, pH = 3.5) [49]. AENM derivatisation is faster (40 °C, 10 min, pH = 7.4), but it requires a lengthy *p*-HMB-based SPE step before derivatisation [44]. AENM-thiol adducts were reported to be stable at room temperature for at least three days [44]. Despite the advantages of stable isotope labelled derivatisations, the reagent pairs have to be synthesised, which could be a potential downside of these approaches.

## 4. Analytical Instrumentation

Appropriate analytical instrumentation and techniques are required for either qualitative or quantitative analysis after thiol extraction and/or derivatisation. GC or LC coupled to various types of detectors, particular MS, are the leading separation and detection techniques used for volatile thiol analysis in wine, foods, and other beverages (Table 2). GC (single or multidimensional) has been coupled with olfactometry (O), flame ionisation detector (FID), flame photometric detector (FPD), pulsed flame photometric detector (PFPD), atomic emission detector (AED), electron-capture detector (ECD), sulfur chemiluminescence detector (SCD), and MS detector (single quadrupole, Q; triple quadrupole, QqQ; ion trap, IT; and, high resolution MS with time-of-flight (TOF, including quadrupole–TOF) or Orbitrap) in the majority of cases. Reversed-phase (RP)–LC conditions coupled with MS detectors (especially QqQ) are the most common configurations for LC-based instrumentation. When using MS detection, EI or CI modes are proposed for GC–MS analysis of thiols, and electrospray ionisation (ESI) in positive mode is frequently reported for the LC–MS methods.

### 4.1. Analysis by GC

GC separations in the gas phase are applicable for volatile analytes, such as thiols and some of their derivatised forms (e.g., PFBBr or ETP derivatives). For injection, a purge and trap injector (PTI) [21] and cool-on-column injection (20 °C [25], 35 °C [25], 40 °C [23]) have been reported for the GC separation of native forms of thiol analytes (e.g., Table 2, Entries 3 and 31). PTI has great extracting and concentrating ability for relatively large volumes of sample (8 mL) [21,56], and cool-on-column injection is preferred for labile analytes [56], hence they are both suitable for the analysis of trace to ultra-trace volatile thiols. The enhancement of thermal stability of analytes after derivatisation allows for the use of conventional injectors and injection modes for thiols, including splitless [18,19,32], large volume (20 µL) [29,30], splitless to split [33,35], TDU in splitless mode [26], and pulsed splitless [28] injection programs. After injection, most of the separations are performed in a one-dimensional GC system installed with fused silica capillary columns, either with non-polar (e.g., BPX-5 [22], DB-5ms [32]) or polar (e.g., HP-Innowax [19], DB-Wax [35]) stationary phases. Selecting the right column for thiol analysis still requires practical trial-and-error approaches, even though multiple options for GC capillary columns are available (Table 2). For example, large volume injection of PFBBr derivatives followed by separation on a VF-5ms column showed problematic chromatographic behaviour (dirty, distorted, broadened, and delayed peaks) and switching to a column with a more polar phase did not resolve this issue [29]. In another instance, peak interferences and tailing when separating PFBBr derivatives on a DB-5 column were overcome by using a DB-FFAP column [33]. In the case of large injection volumes (10 µL), a column with larger internal diameter (0.53 mm i.d.) was preferred [25]. Apart from one-dimensional GC, two-dimensional separations of volatile thiols have also been explored with heart cut GC or GC×GC systems (Table 2, Entries 24, 27, 31) [23,27,57]. However, it was worth noting that, even with the enhanced resolving power of GC×GC, conventional sample preparation procedures without specific chemical derivatisation failed to detect a targeted thiol (4-MSP) due to the high background noise [27].

The detectors of choice in GC applications are normally associated with the analytical aims of the methods; that is, whether for identification or quantitation purposes. O, FID, FPD, PFPD, SCD, and Q-TOF–MS appear to serve as detectors for identification purposes given the sensitivity and selectivity of detectors towards ultra-trace volatile thiols, whereas Q, QqQ, and ITMS are regularly used for quantitation (and can be coupled to GC–O as well). From some of the early work that was focused on screening/discovering volatile thiols in foods and beverages (e.g., Table 2, Entry 1) to as recent as 2017 (e.g., Table 2, Entry 30), GC–O has frequently been utilised to locate odour zones of interest and provide the odour quality of the analytes being isolated [12,21,23]. GC–O also serves as an important criterion for the identification of aroma compounds, and quite remarkably, the human olfactory organ has demonstrated greater sensitivity for certain thiols during GC–O analysis than PFPD or MS [12]. Such ultra-sensitivity towards volatile thiols has been related to specific thiol olfactory receptors (e.g., OR2T11, OR2W1, and OR2C1) in humans [58,59].

FPD [21], PFPD, and SCD [23,34] are important sulfur-selective detectors [65] that are also useful when screening for volatile thiols. These detectors record signal responses of sulfur atoms in the compounds of interest, which provides preliminary chromatographic information. For instance, the retention indices that were calculated upon analysing linear thiol compounds with PFPD have been used for identification purposes (Table 2, Entry 10) [26]. Similar to FPD, SCD has also been applied for screening volatile thiols due to the demonstrated high selectivity and sensitivity (absolute limit of detection below 1 ng, Table 2, Entry 31) [23]. The chromatographic conditions (e.g., GC column and oven program) that are obtained from using detectors such as FPD can then be applied for GC–MS quantitation, as in the case of thiols in soy sauce [66]. Being less expensive to purchase and less complicated to operate and maintain are other practical reasons for using these detectors for routine thiol screening. For thiol quantitation, AED has been evaluated for three volatile thiols in wines (Table 2, Entry 3) and showed good detection performance for 4-MSP, but was not suitable for 3-SH and 3-SHA due to co-elution problems with the IS used [25]. Even so, the LOD of 5 ng/L with AED detection was a few times higher than the ODT of 4-MSP (0.8 ng/L) [25].

MS detectors are still required because of their indispensable ability of obtaining mass spectra for the identification of analytes of interest and the superior quantitation capacity, despite the highly specific coverage of FPD, PFPD, and SCD towards sulfur-containing volatiles. Other than retention index, MS spectra of peaks of interest can be compared to those in commercial databases (e.g., NIST [67] or Wiley [68]), or an in-house thiol database [23]. Accordingly, identity confirmation of new thiols then necessitates the synthesis of reference standards if they are not readily available from commercial suppliers [23,64,69]. The majority of GC-based quantitative analyses are conducted with MS detectors, with electron ionisation (EI) (Table 2, multiple entries) or less frequently used chemical ionisation (CI) (Entries 3, 5, 7, 8, 12) [18,25,29,30,31] being applied for volatile thiol detection. Selected ion monitoring (SIM) has been frequently used over full scan mode, with one quantifier ion and desirably at least two other qualifier ions when single stage MS is applied for quantitative analysis. MS/MS is used in multiple reaction monitoring (MRM), selected reaction monitoring (SRM), or consecutive reaction monitoring (CRM) mode, depending on the detector (QqQ and ITMS), which provides better selectivity and sensitivity than single stage MS. Taking 3-SH, which is probably the most evaluated thiol in wine and beer, as an example, many methods have used MS for its detection. When detected in the native form, SIM ions at *m*/*z* 134, 100, 82, and 67 [19,60] were chosen as the qualifiers and quantifier for single stage MS, whereas transitions *m*/*z* 134→82 and 100→82 were the pairs used for MRM with MS/MS [28]. If 3-SH has been derivatised, ions that were selected in EI–MS have higher *m*/*z*, for instance, 314, 181, and 133 for the PFBBr derivative [32] and 232, 187, and 132 for the ETP derivative [19]. The reported LOD values for 3-SH when using single stage MS were 30 ng/L [32], 69 ng/L [60], 7 ng/L [29], and 2 ng/L [30], which were higher than those values that were obtained with MS/MS (1.9 ng/L [28] and 0.7 ng/L [25]).

### 4.2. Analysis by LC

In recent years, LC-based analytical methods have emerged as promising and novel alternatives for volatile thiol analysis (both screening and quantitation) in foods and beverages (Table 2, Entries 15, 16, 21, 22, 26, 28, 29). Although LC has been used to assess non-volatile thiols, such as cysteine and glutathione, in biological samples for some time [70,71,72], the LC analysis of volatile thiols in foods and beverages has arisen more recently. The high volatility and low abundance of the analytes meant that they were more compatible with GC separations rather than LC with liquid-phase MS detection. Even when bypassing the chromatographic system, underivatised 3-SH was undetectable by direct liquid infusion MS in positive or negative ion mode [43], so to facilitate the potential application of LC to the analysis of volatile thiols, the analytes first have to be converted into non-volatile derivatives with suitable reagents. Indeed, with promising derivatisation reagents having been specifically suggested (Section 3.2.2 and Table 1), complementary LC methods have necessarily been developed in tandem (Table 2, Entries 16, 21, 22, 26, 28), some of which even rival the analytical performance of the GC methods.

RP–LC separations with C18 stationary phases are commonly used for derivatised volatile thiols. The injection volumes and flow rates (0.20–0.40 mL/min) varied slightly, depending on the column and instrument used (Table 2). For example, slightly higher flow rates (0.35 mL/min and 0.40 mL/min) were utilised in ultra-performance (UP) LC with a column containing 1.7 μm particles [36]. Mobile phases are the same as commonly used for RP–LC, for instance, aqueous MeOH [37,43] and aqueous acetonitrile (MeCN) [7,41,44]. Proposed RP–LC methods have been able to resolve the analytes in a relatively shorter period of time (e.g., 17 min with UPLC [36]) compared to GC methods (e.g., 55 min [33]).

MS is still the preferred detection technique for LC methods, even when derivatisation introduces a chromophore into volatile thiols (which may facilitate UV [41] or fluorescence detection [36,45]) (Table 2). In fact, detection is typically undertaken with MS/MS, and instruments, including QqQ, Q-TOF, and Orbitrap operated in ESI mode have been described for identifying or quantitating volatile thiols, with superb sensitivity and selectivity. More specifically, with a QqQ, MRM [7,36,41] is the main mode that is utilised for quantitation and double precursor ion scan (DPIS) mode has been applied more for qualitative purposes [43,49]. The selection of mass transition pairs for MRM typically includes one quantifier pair and at least one qualifier pair as the minimum requirement, with the transitions being chosen from direct infusion product ion MS experiments of the reference standard derivatives [7]. MRM of unique mass transition pairs provides a cleaner chromatographic background, which directly results in greater sensitivity [44]. QqQ with MRM mode has been employed for thiol derivatives that were obtained either with conventional derivatisation reagents [7,33,36,41] or stable isotope derivatisation reagent pairs [44].

DPIS combined with stable isotope labelled derivatisation has been investigated for thiol profiling in beers (Table 2, Entry 16) [43,49]. Admittedly, the thiols that were tentatively identified in beer with this method were biological thiols, but this could be of potential use for volatile thiols analysis. Light- and heavy-labelled thiol derivatives have characteristic ions with a fixed mass shift that can be distinguished by DPIS with QqQ by employing stable isotope labelled derivatisation, and identities can then elucidated by product ion scan and Q–TOF [43]. Only peak pairs from extracted DPIS chromatograms with the same retention time and peak intensity are considered and relative quantitation can be readily achieved by varying the ratios of light- and heavy-labelled samples being mixed [43]. This overall approach could be an attractive option for volatile thiol discovery in foods and beverages.

The application of Orbitrap MS for volatile thiol analysis has been reported for olive oil [37], wine [38], beer [38], and roasted coffee [40]. While using Se-containing ebselen derivatisation, thiol derivatives inherit the selenium isotopic pattern (^80^Se, ^78^Se) and the corresponding accurate masses that were recorded by using Orbitrap MS (mass error tolerance <2 ppm) have been used for tentative identification and quantitation (when thiol reference standards were used) [37]. An Orbitrap MS generated chromatograms with almost no background noise due to its superior sensitivity and selectivity, and the resulting limit of quantitation values were extremely low for 3-SH, at 0.1 ng/kg in olive oil (Table 2, Entry 15) [37], and 0.01 ng/L in wine [38].

### 4.3. LC vs. GC

Overall, the LC methods have been demonstrated to be more sensitive, selective, and faster than GC approaches for quantitative volatile thiol analysis in foods and beverages. Perhaps more importantly, another significant advantage of LC-based methods is that the sample preparation steps are less complicated, markedly so in a number of cases. Furthermore, LC with MS/MS in DPIS mode has potential for untargeted thiol profiling and screening (Table 2, Entry 16). However, as discussed in the next section, the LC–MS approaches have to be treated carefully (particularly for quantitation) to solve analytical challenges from matrix effects [73].

GC approaches generally involve more complicated sample preparation, but are still attractive when compared to LC–MS methods, particularly with respect to the aroma qualities that are obtained by GC–O, which can provide valuable information for thiol identification (or verification) [23] and related sensory studies [74]. In addition, multidimensional GC offers great separation power [75], and with fast GC in the second dimension improving the detection limits and reducing the matrix background, GC×GC coupled with different types of detectors could serve as a powerful tool for discovering new thiols. For instance, GC×GC–TOF–MS has been recently used for thiol screening in wine and fruit, where a total of 11 volatile thiols were identified (Table 2, Entry 31) [23].

GC and LC based methods for thiol analysis are both very versatile and they have great potential in identification and quantitation. The choice of method is often based on instrument availability in the given laboratory, along with capital, operating, and maintenance costs, and most importantly, the analytical methods that are required to address the aims of the research.

### 4.4. Other Instruments

Beyond GC and LC, an ultraperformance convergence chromatography–tandem mass spectrometry (UPC^2^–MS/MS) method has been developed to quantitate volatile thiols in wine [42] after DTDP derivatisation [7]. This proposed method uses supercritical CO_2_ as the primary mobile phase (along with MeOH) at high flow rate (1.5 mL/min), with this convergence chromatography approach providing great efficiency (7 min run time per sample) and being coupled with QqQ (MRM mode) for sensitive detection (with all LODs below the thiol ODT values). Such instrumentation is not common in many laboratories, which may limit application of the method, although this novel approach offers potential high throughput thiol analysis without compromising sensitivity [42].

### 4.5. Matrix Effects and Quantitative Analysis

With the advances in chromatography and MS capabilities, instrumentation with higher separation ability and detection sensitivity has become more available, which leads to a greater number of analytical methods being reported for volatile thiols in more foods and beverages. However, matrix effects in trace analysis have been noted in modern analytical methods that were developed for agricultural samples [76]. In addition, the concentrations of these unstable compounds are at trace to ultra-trace levels and in complex matrices, so their quantitative analysis has to be treated with great care to avoid inaccurate or inconclusive outcomes regarding these potent odour-active molecules.

Matrix effects should be critically evaluated when developing quantitative methods to achieve accurate and reliable results. It is well known that matrix interferences have significant impacts on the extraction, separation, detection, and consequently the quantitation of analytes. This is of particular importance, given both the reactivity and trace concentrations of potent volatile thiols. Matrix effects have been clearly noticed and explored during method development for volatile thiol analysis [18,41]. One way to distinguish the extent of matrix effects is to compare the slopes of calibration curves that have been obtained from different matrices [18,31]. While using this approach, a matrix effect was evidently observed in model wine vs. real wines [18,41] and oxidised vs. non-oxidised wines [30], with various impacts during the extraction, separation, or detection steps potentially leading to differences in the results. For instance, undiluted solvent-assisted flavour evaporation (SAFE) distillate [23], competitive absorption on an HS–SPME fibre [25], or ESI–MS signal enhancement/suppression [77] could cause large matrix effects.

Even when using matrix-matched calibration approaches, the choice of IS can be extremely important in minimising or compensating for matrix effects. Many compounds with similar properties to volatile thiols that showed a negligible matrix effect have been suggested as internal standards, such as 4-methoxy-2-methyl-2-mercaptobutane [22] and 6-sulfanylhexan-1-ol (6-SH) [60]. Even so, stable isotope labelled IS are the best option in a stable isotope dilution assay (SIDA), which is arguably the most accurate analytical approach, and, in most cases, can efficiently eliminate a matrix effect by compensating extraction, separation, and detection variabilities due to the almost identical properties between analytes and their stable isotope, labelled analogues [78]. SIDA has been widely employed for LC–MS [7,36,41] and GC–MS [19,25,27,30,31,32,33] methods for volatile thiols analysis, and both ^2^H [7,19,25,30,32,33,36,41] and ^13^C labelled [27] internal standards have been used in various cases (Table 2, Entries with bolded numbers). Typically, the ideal degree of atom labelling should be greater than two [76], but d_2_-3-SHA [36] and d_2_-3-SH [30,36] have been reported in a few cases as stable isotope labelled IS. Additionally, the elution of stable isotope labelled IS generally occurs slightly earlier than analytes depending on the extent of isotope incorporation, and such examples can be seen for both GC [31,32] or LC [7,41]. However, the co-elution of analytes and stable isotope labelled IS would be desirable to completely compensate for matrix effects [78].

Indeed, SIDA does not guarantee the complete elimination of matrix effects for thiol analysis in wine. It has been shown that deuterated IS spiked into an oxidised wine somehow underwent a faster oxidation than native analyte, which led to a persistent matrix effect [30]. On the other hand, phenols or pigments in red wines have a potentially significant impact on LC–MS/MS signal responses, as evident when comparing calibration curve slopes from different wine and model wine matrices [41]. However, the main limitation with SIDA for thiol analysis is that stable isotope labelled internal standards are often not commercially available or they are expensive to obtain [79]. The same issue applies to stable isotope labelled derivatisation, but it does offer a straightforward and accurate method for quantitative analysis [44] once the reagents have been prepared. Finally, even though stable isotope labelled IS cannot always correct for matrix effects occurring during detection, they are still ideal for overcoming variabilities in extraction, reaction, or adsorptive losses, for instance, which is essential for accurate quantitative analysis.

## 5. Conclusions and Outlook

This review presents an up-to-date overview of the analysis of potent volatile thiols in foods and beverages, with a focus on wine analysis, because that is where many of the methodological advances have arisen. It covers topics from traditional selective extraction, chemical derivatisation (for LC or GC), chromatographic and MS instrumentation, matrix effects, and quantitation considerations. The identification of new volatile thiols and quantitation of known thiols have been made possible over the past three decades thanks to the development of specific thiol extraction methods in combination with sensitive analytical instrumentation. The major observations regarding the current state of potent volatile thiol analysis are: (1) extractions consisting of some form of thiol derivatisation are a popular choices due to their efficiency and simplicity; (2) GC coupled to different detectors (e.g., O, sulfur selective detectors, and MS) are still of considerable utility for discovering new thiol odorants, although newer LC–MS/MS approaches with thiol-specific derivatisation and precursor ion scan experiments also look promising; (3) recently developed quantitative LC–MS methods usually outperform existing GC–MS counterparts when considering the whole protocol, from isolation to analysis; and, (4) SIDA approaches are frequently applied for reliable quantitation.

Undoubtedly, the analysis of potent volatile thiols has been greatly advanced; however, there is still room to further improve the analytical performances to develop faster, more cost-effective, and greener methods that can provide more comprehensive information. In terms of specific extraction, currently available techniques could be coupled with the popular MS detection for the sensitive analysis of volatile thiols. For instance, inspiration could be drawn from the analysis of low molecular weight thiols in water as their *p*-HMB–thiol complexes by LC–ESI–MS/MS after online SPE preconcentration [16]. This offers a stable, sensitive, and selective means for thiol analysis, although the use of mercury features again, and its application to potent volatile thiols in foods and beverages would still need to be investigated. New approaches that apply novel extraction materials should be continuously designed for low cost and effective isolation of volatile thiols, apart from maximising the potential of currently available extraction methods. For example, the potential of novel molecularly imprinted polymer SPME fibres for volatile analysis keeps growing [80], and it seems plausible that SPME fibre coatings could also be customised for volatile thiol extraction. The same can be suggested for SPE sorbent materials, given the recent example of an Ag^+^ based SPE cartridge that has been proposed for volatile thiol analysis [28].

Regarding the future of chromatographic separation in the analysis of volatile thiols, emerging trends include the testing of novel stationary phases and new separation techniques. With respect to the stationary phases, new generation superficially porous silica LC columns have been made commercially available and reported for the separation of a variety analytes [81]. These columns are compatible with conventional HPLC instrumentation (including MS), and the chemistries of the stationary phases are similar to conventional C18 columns, but they offer faster and more efficient separation [81]. Other than new generation LC columns, there are also other stationary phases of potential interest. As an example, a polysaccharide-based chiral LC column has not only demonstrated good analytical performance for separation of thiol enantiomers, but it also revealed the possibility to simultaneously analyse other important achiral volatile thiols [41]. Alternative separation techniques should be also considered, besides improving separation through new columns. The excellent separation efficiency achieved for volatile thiol derivatives by ultraperformance convergence chromatography [42] offers a glimpse of what the future may hold in terms of speed and sensitivity.

The trend for detection is that QqQ and high resolution (Q–TOF or Orbitrap) MS will be more prevalent for both the identification and quantitation of potent volatile thiols. The use of QqQ for LC–MS/MS analyses of thiols has grown strongly in recent years and offers a number of benefits in comparison to GC–MS methods. Future advances should take advantage of the unique fragmentation patterns (with diagnostic fragmented ions) and precursor ion scan mode in LC–MS/MS for the preliminary screening of unknown volatile thiols. Q–TOF and Orbitrap MS detection will also be ideal for such purposes (based on unique isotope pattern of diagnostic ion) due to their unparalleled resolution power and the ability to determine molecular formulas. A few recent reports [37,38,43,49] have explored this non-targeted approach, but ongoing research is required to better answer the complex sensorial, (bio)chemical, and microbiological questions that surround potent thiol odorants in foods and beverages.

## Figures and Tables

**Table 1:** Isolation methods developed for analysis of potent volatile thiols in wines, foods, and other beverages.

Entry No.	Reference	No. of Analytes ^1^	Matrix	Sample Amount	Isolation Overview ^2^	Major Methodological Parameters ^3^	Comments ^4^
1	1995 [21]	1 [ID]	Wine	1000 mL	LLE ⇒ selective extraction [*p*-HMB]	• LLE × 3 using *p*-HMB solution • Glutathione added at 20-fold the *p*-HMB amount	+ Reversible tagging allows thiols to be analysed in native form by GC–O + Suitable for thiol screening with GC based methods − Large sample volume needed − High demand for organic solvents − *p*-HMB is highly toxic − Very time consuming ♦ cheese [26]
2	1998 [22]	5 [QT]	Wine	500 mL	LLE ⇒ selective extraction [*p*-HMB] ⇒ strong anion exchange column ⇒ LLE	• 4-Methoxy-2-methyl-2-butane as IS • pH adjustments • Dowex 1X2-100 column • >45 min for column step	
3	2003 [25]	3 [QT]	Wine	500 mL	LLE ⇒ selective extraction [*p*-HMB]	• SIDA • Wine protected in ice bath under N_2_ • LLE for 15 min • Affi-Gel 501 • 1,4-dithio-dl-threitol (DTT) elution	
4	2017 [27]	1 [QT]	Hops	350 g	LLE ⇒ SAFE ⇒ selective extraction [*p*-HMB] ⇒ SAFE	• SIDA • Mercurated agarose gel prepared from Affi-Gel 10 • LLE duration > 3 h • SAFE at 40 °C • DTT elution • SAFE to remove DTT	
5	2017 [28]	6 [QT]	Beer, hops	20 mL, 2 g	LLE ⇒ Ag^+^ resin based SPE	For beer: • LLE for 15 min • Centrifugation for 15 min • Meta-Sep IC-Ag SPE cartridge • Cartridge reversed • Salted eluate shaken for 15 min • Centrifugation for 15 min	+ Novel SPE concept − Multiple extraction steps
6	2006 [18]	2 [QT]	Wine	10 mL	HS–SPME with automated on-fibre derivatisation [PFBBr]	• PDMS/DVB SPME fibre • Wine bubbled with N_2_ at 4 °C	+ Moderate amounts of sample required + Less solvents needed − Multiple steps for some − Hazardous PFBBr
7	2007 [29]	4 [QT]	Wine	6 mL	LLE ⇒ derivatisation [PFBBr]	• Four IS • LLE with benzene • Sample bubbled with N_2_ • 35 min LLE and centrifugation • PFBBr reaction at 4 °C for 40 min	
8	2008 [30]	5 [QT]	Wine	10 mL	Derivatisation [*o*-methylhydroxylamine for carbonyl of 4-MSP] ⇒ SPE with in-cartridge derivatisation [PFBBr]	• SIDA • Sample purged with N_2_ • *o*-methylhydroxylamine reaction at 55 °C for 45 min • SPE with Bond Elut-ENV	
9	2009 [31]	3 [QT]	Wine	100 mL	Derivatisation [*o*-methylhydroxylamine for carbonyl of 4-MSP] ⇒ SPE with derivatisation [PFBBr] ⇒ HS–SPME	• Similar to [30] • SIDA • DVB/CAR/PDMS for 30 min at 100 °C	
10	2011 [32]	1 [QT]	Wine	200 mL	LLE ⇒ derivatisation [PFBBr] ⇒ HS–SPME	• SIDA • LLE and back extraction with ice-cold aqueous NaOH • PFBBr reaction at room temperature for 20 min • pH adjustment • PDMS/DVB fibre • SPME for 30 min at 80 °C	
11	2015 [33]	3 [QT]	Wine	40 mL	Simultaneous LLE extraction and derivatisation [PFBBr] ⇒ HS–SPME	• SIDA • pH adjustment • PFBBr reaction and LLE for 10 min at room temperature • PDMS/DVB fibre • SPME for 60 min at 70 °C	
12	2013 [19]	3 [QT]	Wine	50 mL	Derivatisation [ETP] ⇒ SPE	• SIDA • pH adjustment • ETP reaction for 10 min under stirring • SPE with ENVI-18	− Poor reaction efficiency with 4-MSP
13	2015 [34]	3 [QT]	Beer, wort, hops	20 mL	Derivatisation [ETP] and SBSE	• SIDA • pH adjustment • PDMS stir bar • ETP reaction for 10 min at 25 °C • NaOH addition • SBSE for 180 min at 1500 rpm	− Long extraction time
14	2014 [35]	1 [QT]	Wine	3 mL	Automated derivatisation of 4-MSP carbonyl [*o*-methylhydroxylamine] and HS–SPME	• SIDA • DVB/CAR/PDMS fibre • SPME for 45 min at 55 °C	+ Easy automated extraction approach − Only one analyte assessed
15	2015 [36]	2 [QT]	Wine	180 mL	LLE ⇒ derivatisation [OPA]	• SIDA • Add potassium metabisulfite and PVPP, stir for 10 min • Centrifugation for 10 min • pH adjustment and sodium borohydride addition • LLE for 20 min • Reaction for 5 min at room temperature	− Large sample volume − Complicated protocol
16	2013 [37]	7 [QT]	Olive oil	2 g	Single step derivatisation [ebselen]	• 4-Methoxy-α-toluenethiol • Reaction maintained under N_2_ • 1 min reaction	+ Simple and fast extraction – Requires high resolution MS ♦ wine [38], beer [38], brewed coffee [39], roasted coffee [40]
17	2015 [7]	5 [QT]	Wine	20 mL	Derivatisation [DTDP] ⇒ SPE	• SIDA • Reaction for 30 min at room temperature • Bond Elut C18 SPE cartridge	+ Simple extraction + Suitable for multiple thiols + Chiral analysis possible ♦ wines [41,42]
18	2014 [43]	1 [ID]	Beer	100 µL	Single step stable isotope labelled chemical derivatisation [d_0_/d_7_-BQB]	• BQB dried under N_2_ • Gly-HCl buffer • Reaction for 1 h at 60 °C	+ Stable isotope derivatisation applied + Precursor ion scan − Synthesis of reagents required
19	2017 [44]	6 [QT]	Wine	100 mL	Selective extraction [*p*-HMB] & SPE ⇒ LLE ⇒ Stable isotope labelled chemical derivatisation [d_0_/d_4_-AENM]	• LiChrolut-EN SPE mercurated with *p*-HMB • Reaction at 40 °C for 10 min	
20	2018 [45]	4 [QT]	Coffee bean, cookies, fried nuts, biscuit	2 g	GP–MSE ⇒ derivatisation [PIPD]	• Gas purge with N_2_ • 1.0 mL of syringe loaded with 0.5 mL of MeOH as extraction solvent • Sample heated for 30 min at 190 °C • Derivatisation for 10 min	+ No pre-enrichment step – Customised extraction apparatus required – Sample subjected to high temp. – Synthesis of reagent required

^1^ ID: identification; QT: quantitation. ^2^ Only major steps are presented. See text and Figure 2 for reagent abbreviations; GP–MSE: gas purge microsyringe extraction. ^3^ IS: internal standard; SAFE: solvent-assisted flavour evaporation; PDMS: polydimethylsiloxane; DVB: divinylbenzene; CAR: carboxen; SIDA: stable isotope dilution assay; PVPP: polyvinylpolypyrrolidone; MeOH: methanol. ^4^ +: advantage; −: disadvantage; ♦: application of similar extraction approaches reported in foods and beverages; GC–O: gas chromatography–olfactory; MS: mass spectrometry.

**Table 2:** Instrumental methods reported for potent volatile thiols in wine, foods, and other beverages.

Entry No. ^1^	No. of Thiols	Year	Matrix	Analyte Form ^2^	Analytical Instrumentation	Aim ^3^	Major Separation Parameters ^4^	ME ^5^	LOD ^6^	RSD ^7^ (%)	Recovery ^8^ (%)
1	1	1995 [21]	Wine	Free	GC–O, –FPD, –EI–MS	ID	• Multiple columns	–	–	–	–
2	5	1998 [22]	Wine	Free	GC–EI–MS	QT	• BP20 (50 m × 0.22 mm, 0.25 µm)	–	–	4–10	75–80
3	3	2003 [25]	Wine	Free	GC–NCI–ITMS/MS, –AED	QT	• DB WAX (30 m × 0.25 mm, 0.5 µm) for MS • DB WAX (30 m × 0.53, 0.5 µm) for AED	–	⇔	<12	–
4	12	2006 [12]	Beer	Free	GC–O, –PFPD, –FID, –EI–MS	ID	• CP-Sil 5 CB (50 m × 0.32 mm, 1.2 µm) or FFAP CB (25 m × 0.32 mm, 0.3 µm) for O, FID, PFPD • DB CP-Sil 5 CB-MS (50 m × 0.32 mm, 1.2 µm) for MS	–	–	–	–
5	2	2006 [18]	Wine	Deriv.	GC–ECD,–NCI–MS	QT	• VF-5ms (20 m × 0.15 mm, 0.15 µm)	Y	<	10–20	–
6	2	2007 [60]	Wine	Free	GC–EI–MS	QT	• INNOwax (30 m × 0.32 mm, 0.25 µm) connected to HP-1 (10 m × 0.32 mm, 0.25 µm)	N	>	–	≈100
7	4	2007 [29]	Wine	Deriv.	GC–CI–MS	QT	• VF-5ms (20 m × 0.15 mm, 0.15 µm)	Y/N	<	10–17	–
8	5	2008 [30]	Wine	Deriv.	GC–CI–MS	QT	• VF-5ms (20 m × 0.15 mm, 0.15 µm)	Y	<	1–20	47–123
9	2	2008 [61]	Wine	Free	GC–EI–MS	QT	• TR-5MS (30 m × 0.25 mm, 0.25 µm)	N	⇔	–	–
10	1	2008 [26]	Cheese	Free	GC–O, –PFPD, –EI–MS	ID, QT	• DB-XLB (30 m × 0.25 mm, 0. 5 µm) • HP-5ms (30 m × 0.25 mm, 0.25 µm)	–	–	–	>70
11	5	2009 [62]	Wine	Free	GC–EI–ITMS	QT	• DB-WAXetr (60 m × 0.25 mm, 0.25 µm)	–	⇔	6.5–12.3	28–123
12	3	2009 [31]	Wine	Deriv.	GC–CI–MS	QT	• Optima Wax (30 m × 0.25 mm, 0.25 µm)	N	<	<10	–
13	1	2011 [32]	Wine	Deriv.	GC–EI–MS	QT	• DB-5ms (60 m × 0.25 mm, 0.25 µm)	N	<	<2.5	–
14	3	2013 [19]	Wine	Deriv.	GC–EI–MS	QT	• HP-INNOwax (60 m × 0.25 mm, 0.25 µm)	N	⇔	1.9–17	94–112
15	7	2013 [37]	Olive oil	Deriv.	HPLC–ESI–Orbitrap MS	QT	• Luna C18 (150 mm × 2.1 mm, 5 µm) • A: 10 mM ammonium formate in water • B: 10 mM ammonium formate in MeOH	Y	<	≈13	79–20
16	1	2014 [43]	Beer	Deriv.	LC–ESI–MS/MS LC–Q–TOF	ID	• VP-ODS column (150 mm × 2.0 mm, 5 μm) • A: 0.1% formic acid in water • B: 0.1% formic acid in MeOH	–	–	–	–
17	1	2014 [35]	Wine	Deriv.	GC–EI–MS/MS	QT	• DB-WAX (60 m × 0.25 mm, 0.25 µm)	N	<	15	99–102
18	3	2015 [33]	Wine	Deriv.	GC–EI–MS	QT	• DB-FFAP (30 m × 0.25 mm, 0.25 µm)	N	⇔	5–11	90–109
19	5	2015 [63]	Wine	Free	GC–MS/MS(QqQ)	QT	• BP20 (2 m × 0.25 mm, 0.22 µm) connected to ZB-1ms (60 m × 0.25 mm, 1 µm)	N	<	5–18	86–110
20	3	2015 [34]	Beer, hops, wort	Deriv.	GC–EI–Q–TOF–MS/SCD GC–EI–MS/MS(QqQ)	ID, QT	• DB-WAX (30 m × 0.25 mm, 0.25 µm) • DB-WAX (15 m × 0.25 mm, 0.25 µm)	–	<	1.3–7.2	99–101
21	2	2015 [36]	Wine	Deriv.	UHPLC–ESI–MS/MS(QqQ)	QT	• Acquity UPLC BEH C18 (100 mm × 2.1 mm, 1.7 μm) • A: 10 mM ammonium acetate in water • B: MeOH:MeCN:isopropanol (49:49:2)	Y/N	<	0.6–11.9	98–128
22	5	2015 [7]	Wine	Deriv.	HPLC–ESI– MS/MS(QqQ)	QT	• Alltima C18 (250 mm × 2.1 mm, 5 μm) • A: 0.5% aqueous formic acid • B: 0.5% formic acid in acetonitrile	–	⇔	<8.5	94–103
23	1	2016 [64]	Wine	Free	GC–EI–MS/MS (QqQ)	QT	• ZB-1ms (60 m × 0.25 mm, 1 µm)	–	<	9	–
24	2	2017 [57]	Wine	Free	GC–EI–MS/MS (QqQ)	ID	• ZB-1ms (60 m × 0.25 mm, 1 µm)	–	–	–	–
25	6	2017 [28]	Beer, hops	Free	GC–EI–MS/MS(QqQ)	QT	• InertCap Pure-WAX (30 m × 0.25 mm, 0.25 µm)	–	<	2.8–8.4	74–113
26	6	2017 [44]	Wine	Deriv.	UHPLC–ESI–MS/MS(QqQ)	QT	• Eclipse Plus C18 column (50 mm × 2.1 mm, 1.8 µm) • A: 0.1% formic acid in 5% aqueous MeCN • B: 0.1% formic acid in MeCN	N	⇔	≤3.5	≥78
27	1	2017 [27]	Hops	Free	GC×GC–Q–TOF Heart cut 2D-GC–CI–ITMS	QT	• Q–TOF: 1st GC: DB-FFAP (30 m × 0.25 mm, 0.25 µm), 2nd GC: DB-5 (2 m × 0.15 mm, 0.30 µm) • ITMS: 1st GC: FFAP (30 m × 0.32 mm, 0.25 µm), cool-on-column injection; 2nd GC: DB-1701 (30 m × 0.25 mm, 0.25 µm)	N	<	<15	109 ± 6 104 ± 4
28	2	2018 [41]	Wine	Deriv.	HPLC–ESI–MS/MS(QqQ)	QT	• Polysaccharide Amylose-1 column (150 mm × 2.0 mm, 3 µm) • A: 5 mM aqueous ammonium bicarbonate • B: MeCN	Y/N	<	<8	90–110
29	8	2018 [45]	Coffee bean, cookie, fried nut, biscuit	Deriv.	HPLC–FLD-APCI-MS	QT	• Eclipse XDB-C18 column (150 mm × 4.6 mm, 5 µm) • A: 30% aq. MeCN • B: MeCN	N	>	4.98	86–97
30	4	2018 [42]	Wine	Deriv.	UPC^2^–MS/MS(QqQ)	QT	• BEH 2-EP column (100 mm × 3 mm, 1.7 µm) • Solvent: CO_2_ and MeOH	N	<	8–18	94–119
31	11	2019 [23]	Fruit, wine	Free	GC–O, –FID, –SCD, GC×GC–Q–TOF	ID, QT	• DB-FFAP (30 m × 0.32 mm, 0.25 µm) • 1st GC: DB-FFAP (30 m × 0.25 mm, 0.25 µm), 2nd GC: DB-17ms (2 m × 0.18 mm, 0.18 µm)	–	–	–	–

^1^ Entry number in bold indicates the method is a stable isotope dilution assay (SIDA). ^2^ free: analytes in free thiol form; deriv.: analytes in thiol derivative form. ^3^ ID: identification, QT: quantitation. ^4^ GC column dimension expressed as (length × internal diameter, film thickness; LC column dimension expressed as (length × internal diameter, particle size); A: mobile phase A; B: mobile phase B; MeCN: acetonitrile; MeOH: methanol. ^5^ ME: matrix effect; Y: ME existed, N: ME not evident; Y/N: ME observed for some analytes; –: not evaluated. ^6^ LOD expressed in comparison to the odour detection thresholds (ODT) of the analytes; <: LOD < ODT; >: LOD > ODT; ⇔: methods involved multiple analytes where LOD > ODT for some analytes and LOD < ODT for others; –: not reported. ^7^ RSD: repeatability (%); –: not reported. ^8^ –: not reported.
